# Mesenchymal Stem Cell–Derived Exosomes Mitigate Cutaneous Radiation Injury Through Coordinated Modulation of DNA Repair, Stress, and Inflammatory Gene Programs

**DOI:** 10.3390/biomedicines14040811

**Published:** 2026-04-02

**Authors:** Amanda Ringwood, Chi Zhang, Rob Knight

**Affiliations:** 1 Cellese Inc., Irvine, CA 92606, USA; 2 Faculty of Medicine, Dentistry and Health Sciences, The University of Melbourne, Carlton, VIC 3053, Australia

**Keywords:** mesenchymal stem cell (MSC), exosome, extracellular vesicle, ionizing radiation, cutaneous radiation injury, skin, dermal fibroblast

## Abstract

**Background**: Cutaneous radiation injury arises when ionizing radiation disrupts epidermal barrier integrity, triggering persistent DNA damage, oxidative stress, and senescence-associated inflammatory signaling that drive extracellular matrix degradation and impaired regeneration. Clinical burden is rising due to dose-intensified radiotherapy, but also due to an increased use of energy-based aesthetic procedures that elicit radiation-like dermal injury. Dermal fibroblasts exhibit marked sensitivity to ionizing radiation and rapidly acquire senescence-associated secretory phenotypes that suppress collagen biosynthesis and promote chronic inflammation, underpinning the need for regenerative treatments that restore tissue homeostasis and regenerative competence. Mesenchymal stem cell–derived exosomes have emerged as a promising therapeutic strategy in this setting, with increasing preclinical evidence demonstrating their capacity to attenuate oxidative stress, enhance DNA damage-repair pathways, and normalize fibroblast metabolic function. **Methods**: In this study, we examine the expression profiles for 14 radiation response–associated genes of irradiated human dermal fibroblasts that were treated with bone marrow and umbilical cord MSC-derived exosomes at different timepoints using quantitative RT-PCR analysis. We also explore functional relationships among these genes through interaction network analysis, and outline a framework to organize pathway-level transcriptional responses to irradiation and exosome treatment. **Results**: MSC-derived exosome treatment was associated with attenuated early damage response signaling at 24 h, followed by increased expression of genes associated with DNA repair and oxidative stress recovery at intermediate timepoints. Exosome-treated cells also exhibited transcriptional changes consistent with modulation of cell-cycle regulatory pathways and reduced expression of pro-inflammatory markers by 5 d. These findings suggest that MSC-derived exosomes influence the temporal organization of the fibroblast transcriptional response to ionizing radiation and may contribute to molecular programs associated with tissue recovery following ionizing radiation exposure.

## 1. Introduction

The skin, as the body’s largest organ and first line of defense, is uniquely susceptible to ionizing radiation (IR) that compromises epithelial integrity, leading to cutaneous radiation injury (CRI) [1,2]. CRI can be classified as either acute or chronic, with acute effects typically manifesting as erythema, desquamation, alopecia, and ulceration that develop within hours to weeks. These symptoms may subsequently progress into chronic effects, including delayed ulcer formation, dermal necrosis, fibrosis, and malignancy that arise over months and years after exposure [2,3,4]. The clinical burden of CRI continues to expand, not only due to increased cancer survivorship and dose-intensified radiotherapy programs, but also through the growing prevalence of energy-based procedures in dermatology and aesthetic medicine, where fractional devices can trigger localized radiation-like tissue responses [1,5,6]. Existing CRI interventions primarily aim to manage patients’ symptoms rather than modify the underlying processes responsible for IR-induced damage, and robust evidence supporting the effectiveness of these treatments is severely lacking [7,8]. These clinical and mechanistic gaps highlight the need for therapies that more directly target the biological consequences of radiation exposure.

Exposure to IR initiates a multi-factorial cascade characterized by complex cellular damage, including DNA double-strand breaks, persistent oxidative stress, cell apoptosis, and the induction of senescence-associated secretory phenotypes (SASPs) that amplify chronic inflammation and extracellular matrix degradation [9,10,11]. Cellular responses to IR involve multiple stress-response pathways. Among the best-characterized are the p53–p21/*CDKN1A* axis, which governs cell-cycle arrest in response to environmental stressors like DNA damage, and NF-κB-mediated signaling that induces production of pro-inflammatory cytokines [12]. Additional pathways, including oxidative stress regulators, DNA damage recognition, and repair mediators, collectively determine whether fibroblasts undergo repair, senescence, or apoptosis [9,13].

Mesenchymal stem cell (MSC)-derived exosomes have recently emerged as a promising approach to mitigate CRI. Exosomes are a class of extracellular vesicles enriched in proteins, lipids, and regulatory microRNAs, capable of transferring functional cargo to recipient cells. Several preclinical studies suggest that MSC-derived exosomes can attenuate oxidative stress, modulate DNA repair capacity, and reduce radiation-induced tissue damage [7,14,15,16,17]. However, the mechanisms by which exosome cargo modulates the transcriptional response of irradiated dermal cells remain incompletely defined. Dermal fibroblasts, which play a critical role in extracellular matrix maintenance and skin integrity, are particularly sensitive to radiation-induced injury, making them a relevant model for studying CRI [18]. Their high susceptibility to IR-induced senescence, collagen loss, and pro-fibrotic SASP signaling is also observed after ablative or fractional energy-based procedures, further supporting their use as a model for radiation-like dermal remodeling [6].

In this study, we investigated the effects of human bone marrow (BM) and umbilical cord (UC) MSC-derived exosomes on irradiated human dermal fibroblasts (HDFs). We performed a targeted qPCR panel to monitor key stress- and DNA-repair-related genes across multiple post-irradiation timepoints (24 h, 3 d, and 5 d). Together, these findings provide insight into the transcriptional programs engaged by MSC-derived exosomes in the context of IR, and highlight their potential as a therapeutic strategy to mitigate CRI.

## 2. Materials and Methods

### 2.1. Primary Cell Culture

Primary human bone marrow mesenchymal stem cells (BM-MSCs) and human umbilical cord mesenchymal stem cells (UC-MSCs) were purchased from RoosterBio, Inc. (SKUs: MSC-031, C43002UC; Frederick, MD, USA). Primary human dermal fibroblasts (HDFs) were purchased from PromoCell GmbH (SKU: C-12302; Heidelberg, Germany). HDF cultures were maintained in DMEM supplemented with 10% fetal bovine serum (FBS; Gibco, Thermo Fisher Scientific, Waltham, MA, USA) at 37 °C with 5% CO_2_. Experiments were performed using HDFs between passages 4 and 6. MSC culture conditions for exosome production are described below in Section 2.2. Exosomes were collected from BM- and UC-MSCs at passage 4.

### 2.2. Exosome Isolation and Characterization

BM- and UC-MSCs were cultured for exosome production in 5-layer cell stacks and allowed to expand for 5 days in xeno-free culture medium (RoosterNourish™-MSC-XF; RoosterBio, Inc.). After 5 days, monolayers were washed with PBS, and the medium exchanged to a particle-free, chemically defined medium for exosome collection (RoosterCollect™-EV; RoosterBio, Inc., Frederick, MD, USA). The medium was conditioned for 48 h before exosomes were isolated by the Exodus H-600 (Exodus Bio, Cambridge, MA, USA) exosome isolation system using a 30 nm pore membrane. Particle concentration was measured by nanoparticle tracking analysis (NTA; Particle Metrix GmbH, Ammersee, Germany). After staining with lipid membrane dye MemGlow™ 488 (Cytoskeleton, Inc., Denver, CO, USA), nano-flow cytometry (NanoFCM Co., Ltd., Nottingham, UK) was used to measure the size and proportion of lipid-based particles. Morphology was assessed by cryo-transmission electron microscopy (cryo-TEM), and direct ELISA was used to detect exosome markers CD9, CD81, LAMP1, ALIX, TSG101, and negative marker calnexin.

To evaluate exosome uptake into fibroblasts, HDF cells were seeded into 8-chamber slides 48 h before the uptake experiment. Exosomes were stained with MemGlow™ 488 and diluted in serum-free DMEM to 4 × 10^10^ particles/mL, a higher exosome particle concentration than that used for treatment experiments, to ensure sufficient fluorescent signal for microscopy and to facilitate visualization of exosome uptake. Cells were incubated with exosomes at 37 °C for 3 h and fixed with 4% paraformaldehyde. Samples were stained with 565-Phalloidin and Hoechst dyes, and covered with Vectashield Vibrance Antifade Mounting Medium (Vector Laboratories, Inc., Newark, CA, USA) before images were taken using an SP8 confocal microscope (Leica Microsystems, Wetzlar, Germany).

### 2.3. Irradiation and Exosome Treatments

HDF cells were seeded into 6-well plates at a density of 4000 cells/cm^2^ (~35,000–40,000 cells/well) and cultured for 48 h prior to exposure to 10 Gy X-ray radiation. After irradiation, cells were treated with exosomes isolated from BM-MSCs or UC-MSCs. The treatment media contained 10% *v*/*v* exosomes in PBS for a final concentration of 1 × 10^9^ particles/mL in DMEM with 2% FBS. Exosome-treated groups were compared against irradiated and non-irradiated Vehicle controls (10% *v*/*v* PBS). Cells were treated for three timepoints, 24 h, 3 d, and 5 d post-irradiation, with treatment media changes every other day.

### 2.4. Quantitative RT-PCR (qPCR)

RNA was extracted from HDFs using the RNeasy Mini Kit (Qiagen, Hilden, Germany), according to the manufacturer’s instructions. RNA concentration was measured by UV–Vis spectroscopy using a NanoPhotometer N60 (Implen GmbH, Munich, Germany). qPCR was conducted using UltraPlex 1-Step ToughMix (Quantabio, LLC, Beverly, MA, USA) and TaqMan Gene Expression Assay primers (Thermo Fisher Scientific Inc., Waltham, MA, USA; see Table 1) on a QuantStudio 5 RealTime PCR System (Quantabio, LLC). Each reaction contained 50 ng of RNA, and all samples were analyzed in three technical triplicates per gene. Relative gene expression was calculated using the comparative Ct (∆∆Ct) method, with *GAPDH* as the reference gene and the non-irradiated Vehicle group at the corresponding timepoint as the reference condition.

### 2.5. Interaction Network Analysis

Genes in the qPCR panel were imported into Cytoscape 3.10.4, and a functional interaction (FI) network was generated using ReactomeFI 8.0.10 [19]. Network construction was performed with default settings and linker genes enabled to connect network components. The resulting network was visualized and organized into functional groupings based on known biological roles relevant to the IR response.

### 2.6. Statistical Analysis

Statistical analysis and data visualization were performed using GraphPad Prism 10.6.0 (GraphPad Software LLC, San Diego, CA, USA). Gene expression of experimental groups was compared using ordinary one-way ANOVAs with a significance level of *p* < 0.05. Post hoc multiple comparisons were conducted using Dunnett’s test to compare treatment conditions against the timepoint-matched Vehicle-IR group. All data are presented as mean ± standard deviation (SD), and all error bars show SD.

## 3. Results

### 3.1. Exosome Characterization and Uptake by Human Dermal Fibroblasts

**Figure 1:**
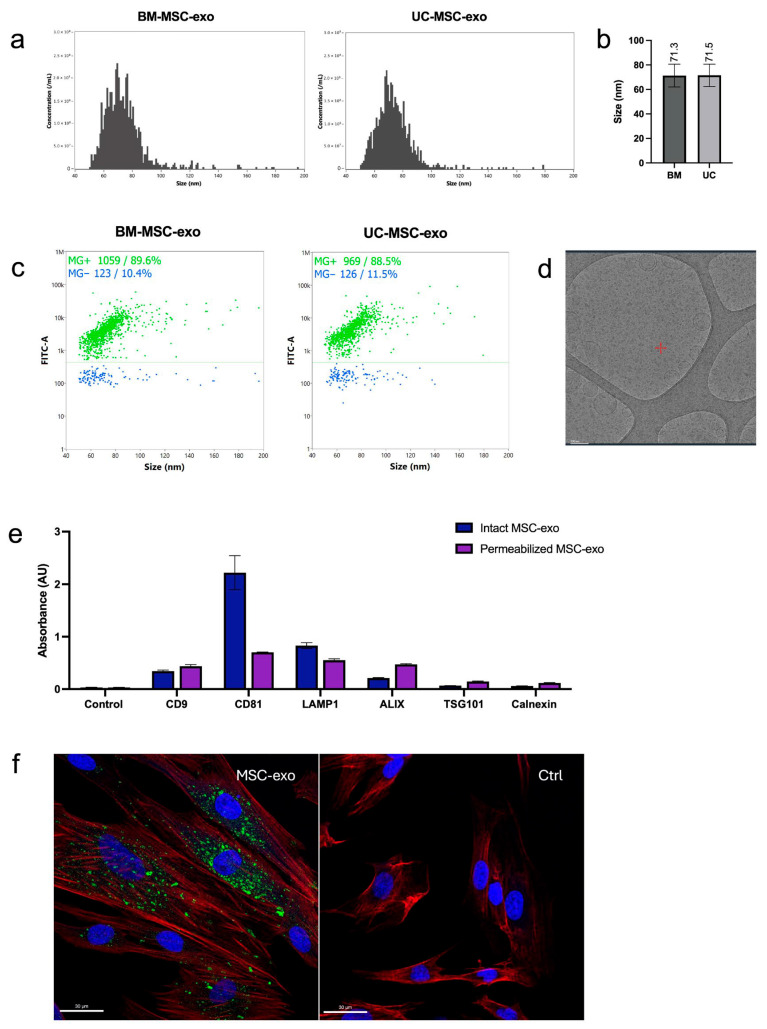
Characterization of BM- and UC-MSC-derived exosomes (BM-MSC-exo; UC-MSC-exo): (**a**) Size distributions, (**b**) mean sizes, and (**c**) percentage of lipid-based particles of BM-MSC-exo and UC-MSC-exo determined by NanoFCM. MG+ = lipid-positive particles stained with MemGlow 488; MG− = lipid-negative particles. (**d**) TEM image of 1:1 mix of BM-MSC-exo and UC-MSC-exo, depicting standard exosome morphology. (**e**) Detection of exosome markers of 1:1 mix of BM-MSC-exo and UC-MSC-exo by direct ELISA. Surface markers (CD9, CD81) and luminal markers (LAMP1, ALIX, TSG101) were measured in intact (non-permeabilized) and permeabilized exosomes to distinguish membrane-associated and internal proteins, with calnexin as a negative marker. (**f**) Confocal images of human dermal fibroblasts, confirming uptake of 1:1 mix of BM-MSC-exo and UC-MSC-exo. Red = F-actin; blue = cell nucleus; green = exosomes.

BM-MSC- and UC-MSC-derived exosomes (BM-MSC-exo and UC-MSC-exo) were purified from conditioned media to concentrations of 7.7 × 10^11^ particles/mL and 5.9 × 10^11^ particles/mL, respectively, as measured by NTA. Nano-flow cytometry demonstrated that both particle solutions displayed single-peak size distributions (BM-MSC-exo: 71.3 ± 9.3 nm, UC-MSC-exo: 71.5 ± 9.1 nm) that fall into the expected size range of exosomes, between 30 and 150 nm (Figure 1a,b) [20]. After staining the particles with MemGlow™ 488, a lipid membrane dye, high proportions of lipid-based particles were confirmed: 89.6% for BM-MSC-exo and 88.5% for UC-MSC-exo (Figure 1c). To assess cellular uptake, BM-MSC-exo and UC-MSC-exo were mixed 1:1, stained with MemGlow™ 488, and incubated with human dermal fibroblasts (HDFs) for 3 h. Internalization of MSC-exo into HDFs was verified by confocal imaging (Figure 1d).

**Figure 2:**
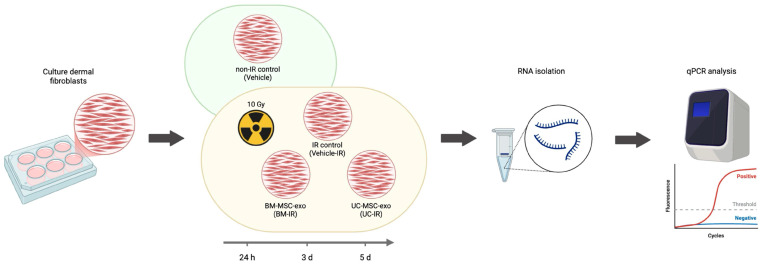
Schematic of experimental design. Human dermal fibroblasts are cultured in 6-well plates, then divided into experimental groups: non-irradiated control (Vehicle) and IR groups irradiated with 10 Gy X-rays—IR control (Vehicle-IR), BM-MSC-exo treatment (BM-IR), and UC-MSC-exo treatment (UC-IR). Cells are treated for three timepoints (24 h, 3 d, 5 d), and RNA is isolated before performing qPCR analysis to compare gene expression patterns across experimental groups. Created in BioRender. Knight, R. (2026) https://BioRender.com/k0k1o5l, accessed on 18 March 2026.

To evaluate the effects of MSC-exo on CRI, irradiated HDFs (10 Gy) were treated with BM-MSC-exo or UC-MSC-exo (treatment groups: BM-IR and UC-IR) at a concentration of 1 × 10^9^ particles/mL, and compared against untreated, irradiated HDFs (Vehicle-IR) and untreated, non-irradiated HDFs (Vehicle) (Figure 2). Quantitative RT-PCR analysis was performed on the RNA extracted from cells that were treated for three timepoints: 24 h, 3 d, and 5 d post-irradiation. A qPCR panel of 14 genes was assembled to cover different functional categories relevant to CRI, including DNA damage and stress response, cell-cycle regulation, and inflammation.

### 3.2. MSC-Exo Dampen Early DNA Damage Signaling and Stress-Response Gene Expression

**Figure 3:**
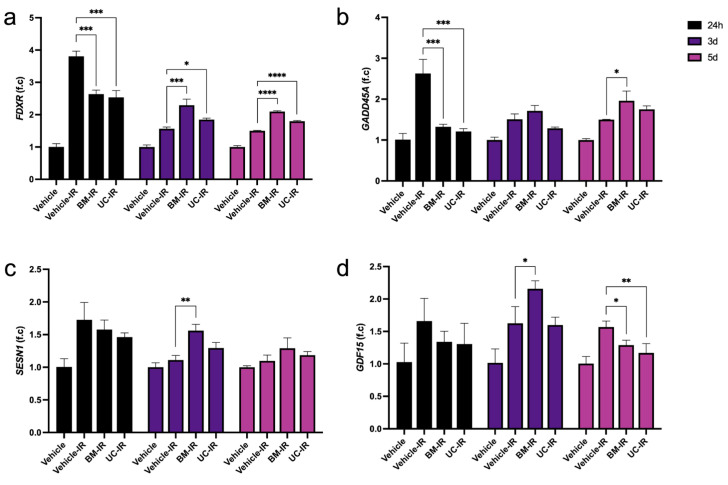
Expression of early damage and stress-response genes (**a**) *FDXR*, (**b**) *GADD45A*, (**c**) *SESN1*, and (**d**) *GDF15* in irradiated HDFs (10 Gy) treated with exosomes (1 × 10^9^ particles/mL) derived from BM-MSCs (BM-IR) and UC-MSCs (UC-IR), measured by qPCR. Gene expression was normalized to *GAPDH* and expressed as fold change relative to the non-irradiated Vehicle control at each timepoint. Data are shown as mean ± SD of three technical replicates from a representative experiment performed using a single HDF donor. * *p* < 0.05, ** *p* < 0.01, *** *p* < 0.001, **** *p* < 0.0001 by ordinary one-way ANOVA followed by Dunnett’s post hoc test, comparing each treatment group to the Vehicle-IR control at the corresponding timepoint.

*FDXR* is a p53 downstream target well-known for its rapid dose-dependent response to IR exposure, making it a reliable estimator of IR dosage [21]. At 24 h post-IR exposure, *FDXR* transcript levels were upregulated nearly four-fold in the Vehicle-IR group compared to the non-irradiated Vehicle control, confirming expected activation of the early radiation-damage response (Figure 3a). Both MSC-exo-treated groups (BM-IR and UC-IR) also displayed increased *FDXR* expression relative to the Vehicle control group, but their levels remained 1.4-fold lower than the Vehicle-IR group (*p* < 0.05). This attenuation indicates that MSC-exo treatment was associated with a reduced magnitude of early p53 stress gene induction.

By 3–5 d post-IR, *FDXR* expression in the Vehicle-IR group had declined from the 24 h peak, consistent with the expected resolution of acute stress signaling. In contrast, both BM-IR and UC-IR groups exhibited higher *FDXR* levels at these timepoints, surpassing the Vehicle-IR group. This divergence from the early-timepoint trend suggests that while MSC-exo dampened the immediate *FDXR* peak, it may reflect a delayed or prolonged transcriptional response involving mitochondrial and p53-associated stress genes at 3–5 d.

*GADD45A*, another p53-regulated gene, induces cell-cycle arrest at the G_2_/M checkpoint in response to genotoxic stress, allowing the recruitment of DNA repair mechanisms or initiation of apoptosis in cases of severe damage [22,23,24]. *GADD45A* was observed to follow a similar biphasic pattern to *FDXR*. *GADD45A* levels peaked at 24 h in the Vehicle-IR group, indicating activation of the downstream DNA damage response, but the BM-IR and UC-IR groups showed a ~0.5-fold lower expression than the Vehicle-IR group at this early timepoint, while by 3–5 d, the MSC-exo-treated groups had slightly higher or nearly equal expression compared to the Vehicle-IR group (Figure 3b). These findings are consistent with attenuation of early stress-response gene expression after 24 h of MSC-exo treatment following IR exposure.

Early spikes in other stress-responsive genes, *SESN1* and *GDF15*, were also observed at 24 h for all IR groups compared to the Vehicle control (Figure 3c,d). However, at 3 d, BM-IR expresses significantly higher levels of both genes than the Vehicle-IR group. Both *SESN1* and *GDF15* are induced by p53 and promote cell survival after oxidative stress, though by different mechanisms [25,26]. At 5 d, *SESN1* expression nearly normalized to the non-irradiated Vehicle baseline with no significant difference between any of the irradiated groups. However, *GDF15* expression in both BM-IR and UC-IR was reduced to <0.8-fold of the Vehicle-IR group at 5 d. The return of *SESN1* to baseline and the continued reduction in *GDF15* in MSC-exo-treated cells may suggest a potential resolution of stress-response transcriptional activity.

Across *FDXR*, *GADD45A*, *SESN1*, and *GDF15*, a shared temporal pattern emerged: MSC-exo cells displayed reduced early (24 h) stress gene activation but enhanced mid-phase (3–5 d) expression, suggesting that MSC-exo treatment is associated with reduced early stress gene induction and altered temporal expression of genes linked to cellular adaptation during the recovery phase.

### 3.3. MSC-Exo Treatment Is Associated with Increased Expression of Genes Involved in DNA Repair and Oxidative Stress Reduction During the Intermediate Phase

**Figure 4:**
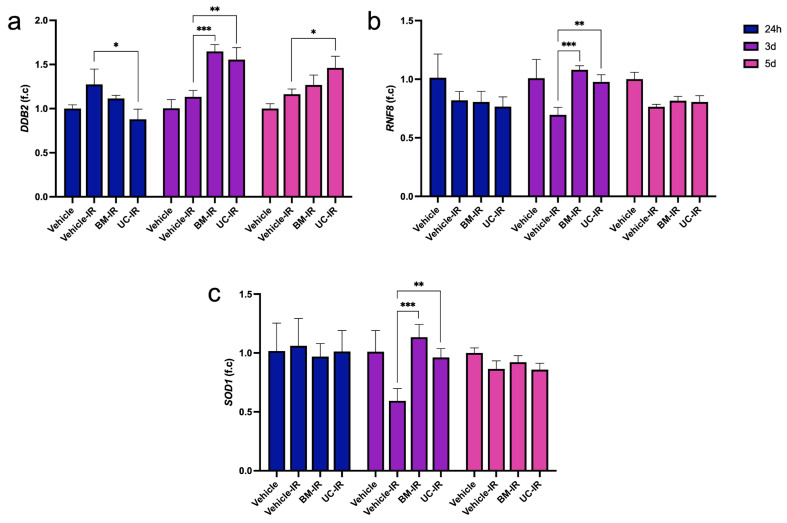
Expression of DNA repair and antioxidant genes (**a**) *DDB2*, (**b**) *RNF8*, and (**c**) *SOD1* in irradiated HDFs (10 Gy) treated with exosomes (1 × 10^9^ particles/mL) derived from BM-MSCs (BM-IR) and UC-MSCs (UC-IR), measured by qPCR. Gene expression was normalized to *GAPDH* and expressed as fold change relative to the non-irradiated Vehicle control at each timepoint. Data are shown as mean ± SD of three technical replicates from a representative experiment performed using a single HDF donor. * *p* < 0.05, ** *p* < 0.01, *** *p* < 0.001, by ordinary one-way ANOVA followed by Dunnett’s post hoc test comparing each treatment group to the Vehicle-IR control at the corresponding timepoint.

IR exposure activated the expression of several genes involved in genome maintenance and oxidative stress resilience, with expression peaking mostly at 3 d. *DDB2*, a key regulator of global nucleotide-excision repair, was significantly upregulated in BM-IR and UC-IR at 3 d (1.5-fold and 1.4-fold, respectively) and UC-IR (1.3-fold) at 5 d relative to the Vehicle-IR group at the corresponding timepoint (Figure 4a). *RNF8*, an E3 ubiquitin ligase required for recruitment of downstream repair factors to DNA double-strand breaks, was similarly elevated in both the BM-IR and UC-IR groups at the 3 d timepoint (Figure 4b), suggesting involvement of ubiquitin-dependent chromatin remodeling processes linked to double-strand break repair. These findings support a coordinated DNA damage response involving both nucleotide excision repair and double-strand break repair pathways.

The antioxidant enzyme encoded by *SOD1*, which catalyzes the dismutation of superoxide radicals, also showed increased gene expression following radiation. Like *DDB2* and *RNF8*, *SOD1* levels were highest at 3 d in the BM-IR and UC-IR groups relative to the Vehicle-IR group (Figure 4c). All groups had normalized to the non-irradiated Vehicle group by 5 d. The 3 d peak in *SOD1* expression may reflect a compensatory redox-protective response to oxidative stress following IR. The combined induction of *DDB2*, *RNF8*, and *SOD1* in MSC-exo-treated groups suggests coordinated transcriptional regulation of genes associated with genome maintenance and oxidative stress responses.

### 3.4. MSC-Exo Treatment Is Associated with Transcriptional Changes Related to Cell-Cycle Regulation, Proliferation, and Angiogenesis After IR

**Figure 5:**
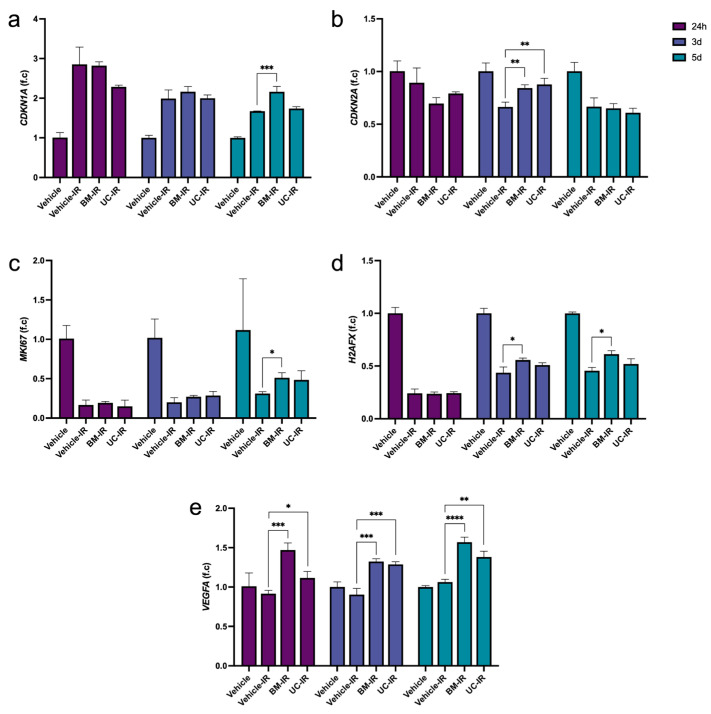
Expression of cell-cycle regulation and proliferation genes (**a**) *CDKN1A*, (**b**) *CDKN2A*, (**c**) *MKI67*, (**d**) *H2AFX*, and (**e**) *VEGFA* in irradiated HDFs (10 Gy) treated with exosomes (1 × 10^9^ particles/mL) derived from BM-MSCs (BM-IR) and UC-MSCs (UC-IR), measured by qPCR. Gene expression was normalized to *GAPDH* and expressed as fold change relative to the non-irradiated Vehicle control at each timepoint. Data are shown as mean ± SD of three technical replicates from a representative experiment performed using a single HDF donor. * *p* < 0.05, ** *p* < 0.01, *** *p* < 0.001, **** *p* < 0.0001 by ordinary one-way ANOVA followed by Dunnett’s post hoc test, comparing each treatment group to the Vehicle-IR control at the corresponding timepoint.

The cyclin-dependent kinase inhibitors *CDKN1A* (p21) and *CDKN2A* (p16) are involved in activating different checkpoint pathways that halt cell-cycle progression to allow time for DNA repair [9,12]. p21 specifically mediates G_1_/S arrest downstream of p53, while p16 controls the G_1_/S transition via the inhibition of CDK4/6 to maintain cells in a senescent-like state, and is associated with long-term growth arrest [9,11]. Contrasting dynamics between *CDKN1A* and *CDKN2A* expression were observed. *CDKN1A* was upregulated in all IR groups relative to the non-irradiated Vehicle group, in line with its established role in mediating temporary cell-cycle arrest at the G_1_/S checkpoint following IR exposure (Figure 5a). *CDKN2A*, in contrast, showed downregulated or normalized expression in the IR groups compared to the non-irradiated Vehicle group (Figure 5b). At 3 d, both the BM-IR and UC-IR groups expressed significantly higher levels of *CDKN2A* than the Vehicle-IR group, coinciding with the increase in expression of DNA repair genes also observed at 3 d. Upregulation of both *CDKN1A* and *CDKN2A* is consistent with transcriptional signatures associated with checkpoint activation following DNA damage that limits propagation of damaged cells.

Conversely, markers of proliferation showed distinct patterns that may potentially indicate ongoing recovery. *MKI67*, which is expressed only in actively cycling cells, was expectedly reduced in the IR groups relative to the non-irradiated Vehicle group (Figure 5c). Over 3–5 d, *MKI67* expression in MSC-exo-treated groups slowly increased, and at 5 d, *MKI67* levels in BM-IR were significantly higher (1.6-fold) than in the Vehicle-IR group. Expression of *H2AFX*, encoding histone protein H2AX whose phosphorylated form (γ-H2AX) serves as a standard marker for the presence of DNA double-strand breaks, mirrors that of *MKI67*, with steep drops in expression observed at 24 h in all IR groups, while cell-cycle progression may stop to repair IR-induced DNA damage, and the moderate rise in expression at 3–5 d for the BM-IR group point towards resuming regular transcriptional activity (Figure 5d) [27]. These transcriptional patterns may align with partial recovery of HDF proliferative capacity by 5 d post-IR exposure, most notably in the BM-IR group.

In addition, *VEGFA*, a primary driver of angiogenesis, displayed significantly higher expression in both MSC-exo-treated groups than the Vehicle-IR group across all timepoints (Figure 5e). BM-IR demonstrated a stronger stimulatory effect on *VEGFA* expression than UC-IR, where expression increased 1.60-fold relative to that from the Vehicle-IR group for BM-IR versus 1.22-fold for UC-IR at 24 h, as well as a 1.48-fold increase for BM-IR versus a 1.30-fold increase for UC-IR at 5 d. Together, these results suggest that transcriptional changes associated with pro-angiogenic pathways occur following MSC-exo treatment in irradiated fibroblasts, with a trend toward stronger induction observed in BM-IR compared to UC-IR.

### 3.5. IR Triggers an Inflammatory Response That Is Attenuated by MSC-Exo by 5 d

**Figure 6:**
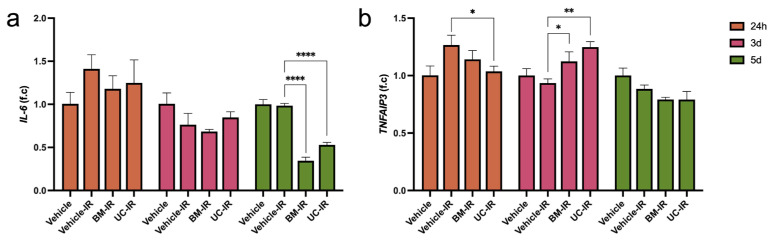
Expression of inflammatory response genes (**a**) *IL-6* and (**b**) *TNFAIP3* in irradiated HDFs (10 Gy) treated with exosomes (1 × 10^9^ particles/mL) derived from BM-MSCs (BM-IR) and UC-MSCs (UC-IR), measured by qPCR. Gene expression was normalized to *GAPDH* and expressed as fold change relative to the non-irradiated Vehicle control at each timepoint. Data are shown as mean ± SD of three technical replicates from a representative experiment performed using a single HDF donor. * *p* < 0.05, ** *p* < 0.01, **** *p* < 0.0001 by ordinary one-way ANOVA followed by Dunnett’s post hoc test, comparing each treatment group to the Vehicle-IR control at the corresponding timepoint.

Expression of *IL-6*, a pro-inflammatory cytokine and primary SASP factor involved in the acute-phase immune response, was upregulated in IR groups at 24 h compared to the Vehicle group, as expected following IR exposure (Figure 6a). By 5 d, *IL-6* levels remained elevated in the Vehicle-IR group, similar to the non-irradiated Vehicle, but were significantly downregulated in the BM-IR and UC-IR groups, decreasing to 0.35-fold and 0.54-fold of Vehicle-IR levels, respectively. *TNFAIP3*, a negative regulator of NF-κB inflammatory signaling, also showed significantly higher expression in the BM-IR and UC-IR groups (1.20-fold and 1.33-fold, respectively) relative to the Vehicle-IR group at 3 d (Figure 6b). These findings demonstrate a potential association between MSC-exo treatment and reduced expression of pro-inflammatory markers, as well as a matching increase in expression of genes involved in negative regulation of NF-κB signaling.

### 3.6. Integrated Network Analysis Reveals Coordinated Radiation-Response Pathways

**Figure 7:**
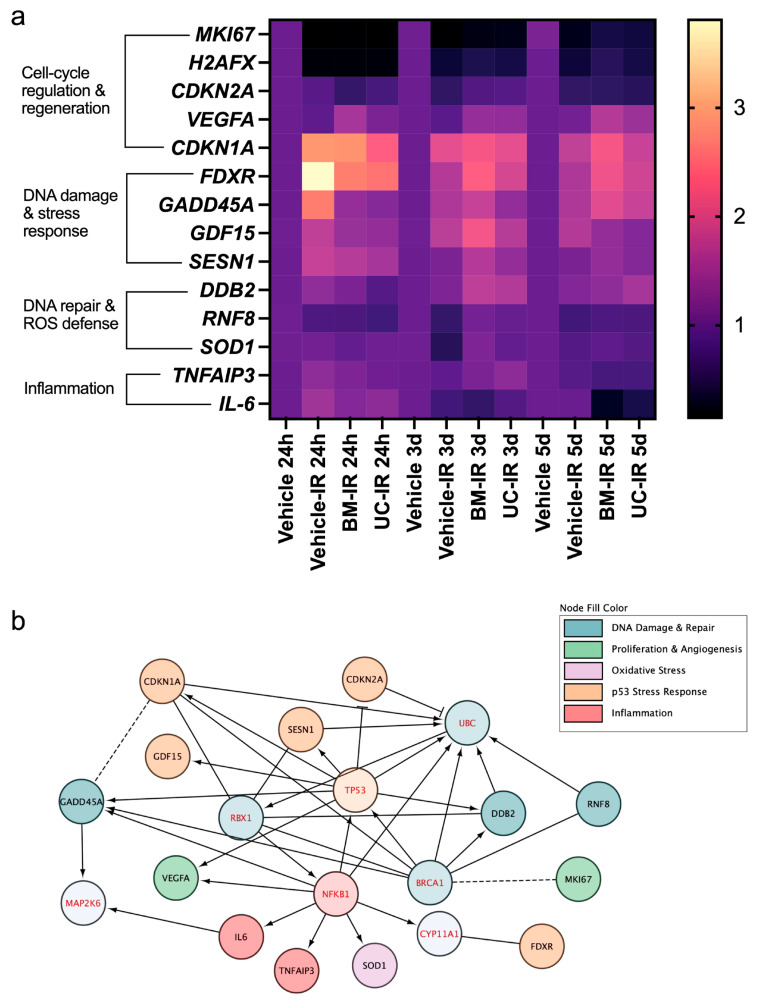
Integrated heat map and interaction network analysis of radiation-responsive transcriptional programs in irradiated human dermal fibroblasts: (**a**) Heat map summarizing relative gene expression changes across all experimental conditions and timepoints (24 h, 3 d, and 5 d post-irradiation). Expression values represent mean log2-transformed relative quantification (RQ) normalized to *GAPDH* and scaled per gene to highlight temporal and treatment-associated expression patterns. (**b**) Functional interaction network constructed using the ReactomeFI plugin in Cytoscape based on genes included in the qPCR panel. Nodes represent individual genes (black text = genes in qPCR panel; red text = linker genes not in qPCR panel). Edges indicate curated functional interactions derived from Reactome pathway knowledge (solid edges = direct or well-established interactions; dashed edges = predicted interactions).

To contextualize the gene-level changes described above, we generated an integrated expression heat map summarizing all IR-responsive transcripts across treatment conditions and timepoints (Figure 7a). This visualization revealed time-dependent transcriptional patterns across the gene panel following irradiation. MSC-exo-treated groups exhibited coordinated differences in the expression of multiple genes relative to the Vehicle-IR control group, potentially reflecting modulation of key pathways involved in stress response, repair, cell-cycle regulation, and inflammation.

Notably, genes associated with early stress signaling and inflammation showed strong induction at 24 h following IR, whereas genes involved in DNA repair, antioxidant defense, and proliferative recovery displayed more heterogeneous temporal regulation over the 5 d experimental duration. MSC-exo-treated samples demonstrated a more dynamic transcriptional trajectory across timepoints compared to the Vehicle-IR group, which may indicate a regulated transition from early damage response toward recovery-associated gene expression programs.

Genes associated with early stress and p53-mediated signaling (*FDXR*, *GADD45A*, *SESN1*, *GDF15*, and *CDKN1A*) displayed elevated expression at 24 h post-irradiation, followed by an overall gradual attenuation at later timepoints, aligning with potential resolution of acute damage signaling and transition toward repair and recovery phases. In contrast, transcripts involved in DNA repair and redox homeostasis (*DDB2*, *RNF8*, and *SOD1*) showed later induction, with peak expression occurring predominantly at 3 d, reflecting transcriptional activity related to genome maintenance and oxidative stress defense during the intermediate response phase.

Cell cycle–associated genes and proliferative markers (*CDKN2A*, *H2AFX*, *MKI67*, and *VEGFA*) exhibited distinct temporal profiles characterized by primarily early suppression followed by partial normalization or increased expression at later timepoints, suggesting checkpoint activation and subsequent cues for proliferative recovery and tissue remodeling. Inflammatory mediators (*IL-6* and *TNFAIP3*) formed a separate expression pattern marked by early induction and subsequent regulation over time, suggestive of an acute inflammatory response with engagement of negative feedback mechanisms. Across these functional groupings, MSC-exo treatment conditions demonstrated coordinated differences in temporal expression patterns relative to the Vehicle-IR control, supporting a modulatory influence of exosome treatment on the integrated cellular response to irradiation.

To provide pathway context for the genes assessed, interaction network analysis was performed using the ReactomeFI plugin in Cytoscape (Figure 7b). The resulting functional interaction (FI) network illustrates a highly interconnected structure linking stress response, DNA repair, oxidative defense, cell-cycle regulation, and inflammatory signaling pathways based on previously described functional relationships. Central nodes such as *TP53*, *NFKB1*, *BRCA1*, *RBX1*, and UBC served as hubs connecting multiple IR-responsive genes. Peripheral hub nodes include *MAP2K6*, which was connected with other early damage signaling genes (*GADD45A* and *IL-6*), consistent with its known role in transducing DNA damage and oxidative stress signals via p38 MAPK signaling, and *CYP11A1*, which connected *FDXR* with the NF-κB pathway, reflecting *FDXR*’s known involvement in broader metabolic or mitochondrial adaptations to irradiation downstream of canonical DNA damage signaling, rather than a direct role in DNA lesion recognition or repair [28].

When considered alongside the gene expression data, the FI network provides a framework for interpreting how the genes examined relate to multiple interconnected biological pathways rather than a single functional category. Together, the heat map and network visualizations provide contextual interpretation of the transcriptional response to ionizing radiation within known biological pathways, and illustrate how MSC-derived exosome treatment is associated with coordinated expression patterns across genes involved in damage response, repair, inflammation, and tissue recovery.

## 4. Discussion

Cutaneous radiation injury persists as a significant clinical challenge due to the prevalence of cancer radiotherapy treatments as well as the increasing use of fractional devices in aesthetic medicine. However, current treatments for CRI are largely focused on alleviating symptoms instead of restoring tissue function. Beyond inflammatory and vascular damage, ionizing radiation induces persistent DNA lesions and genomic instability that drive fibroblast senescence and sustained loss of tissue regenerative capacity. Therapeutic strategies capable of supporting endogenous DNA damage response and repair pathways therefore represent a critical unmet need for both functional tissue recovery and long-term skin health. Many studies have been conducted on the potential of mesenchymal stem cell–based therapies, but MSC-derived exosomes have recently garnered interest because of their improved stability, circulation half-life, and ability to cross biological barriers while still conferring similar benefits to MSCs themselves [2,29]. MSC-derived exosomes have been shown to have positive effects on inflammation, angiogenesis, and cell survival, but current research exploring their application to CRI still remains fairly limited [29].

Exosomes sourced from adipose-derived stem cells (ADSCs) have been demonstrated to improve erythema, desquamation, and fibrosis in rodent CRI models, with tissue sections revealing an increased number of blood vessels and decreased expression of pro-inflammatory cytokines, such as *IL-6* and *IL-1β* [7,14]. Dermal fibroblasts treated with ADSC-derived exosomes in vitro also displayed lower intracellular ROS, higher rates of cell proliferation, and elevated gene expression of growth factors, including *VEGF* [7,14]. Exosomes derived from other MSC sources as possible treatments for CRI have not yet been investigated, although bone marrow MSC-derived exosomes have been shown to alleviate radiation-induced bone loss by promoting DNA repair of double-strand breaks, reducing expression of cell senescent proteins, and activating Wnt/β-catenin signaling in recipient BM-MSCs [16]. Notably, impaired DNA damage repair and accumulation of senescent cells are also central hallmarks of cutaneous aging, linking radiation injury to broader mechanisms of skin degeneration. These observations suggest that MSC-derived exosomes may exert benefits that extend beyond acute radioprotection, potentially influencing long-term tissue resilience and longevity-associated pathways in skin. Outside the radiation context, umbilical cord MSC-derived exosomes have been demonstrated to reduce oxidative stress damage and increase viability of renal epithelial cells exposed to calcium oxalate crystals—the primary compound found in kidney stones [30]. These findings support the rationale for exploring BM- and UC-derived exosomes as distinct MSC sources with potentially complementary mechanisms of action in the context of CRI.

In the present study, we investigated how BM- and UC-MSC-derived exosomes influence the transcriptional response of irradiated human dermal fibroblasts across early and intermediate post-irradiation timepoints. Using a targeted qPCR panel of genes spanning DNA damage response, repair, cell-cycle regulation, and inflammation, we observed that irradiation elicited a stress response consistent with established models of radiation injury. Previously, studies have shown that IR exposure upregulates DNA damage and stress-response genes *FDXR*, *CDKN1A*, and *SESN1* across multiple cell types, while severely limiting dermal fibroblast proliferative capacity, including reduced expression of *MKI67* [13,21,31]. Our results were consistent with these established transcriptional patterns.

We also found that MSC-exosome treatment was associated with structured modulation of gene expression dynamics rather than complete suppression of radiation-induced signaling. MSC-exosome-treated HDFs exhibited attenuated expression of early stress-associated genes compared to irradiated control groups at 24 h, but this was followed by increased expression of those early stress genes as well as other genes associated with DNA repair, ROS protection, proliferation, and anti-inflammatory signaling at 3–5 d. The differences observed between early and later timepoints may reflect the transition from acute damage-recognition signaling toward repair- and recovery-associated transcriptional programs following irradiation. Interaction network analysis demonstrated that the MSC-exosome-associated transcriptional changes we observed align with known stress-response pathways—centered on hubs such as *TP53*, *RBX1*, *UBC*, and *NFKB1*—supporting the interpretation that these responses may reflect coordinated pathway regulation rather than isolated gene expression modulation. These findings suggest that MSC-derived exosomes may influence the temporal organization of the fibroblast radiation response, potentially promoting transcriptional profiles that support cellular recovery while preserving early damage-recognition signaling.

Rather than directly contrasting efficacy between BM- and UC-MSC sources, our findings suggest that exosomes from distinct MSC origins may activate overlapping yet context-dependent protective pathways. While prior studies have demonstrated protective effects of ADSC-derived exosomes in rodent CRI models, the present work extends this research by showing that BM- and UC-derived MSC exosomes were associated with coordinated time-dependent transcriptional programs in irradiated dermal fibroblasts. Although these findings provide insight into the organization of MSC-exosome-mediated radiation responses, additional studies will be required to define the functional consequences and molecular determinants underlying these effects. From a translational perspective, the ability of MSC-derived exosomes to influence DNA damage response and repair pathways may be particularly relevant to longevity-focused and regenerative aesthetic applications, where preservation of genomic integrity, cellular function, and regenerative capacity are increasingly recognized as foundational drivers of durable clinical outcomes.

Several considerations should be taken into account when interpreting these findings. First, experiments were conducted using primary human dermal fibroblasts derived from a single donor source. While the use of primary cells provides physiologically relevant responses, fibroblast populations can exhibit donor-dependent variability in radiation sensitivity and stress-response signaling. Future studies incorporating fibroblasts from multiple donors will, therefore, be important to determine the extent to which the transcriptional patterns observed here are broadly conserved. Second, the 10 Gy radiation dose used in this study represents a relatively high experimental dose compared with conventional fractionated clinical radiotherapy, but it was selected to reliably induce robust DNA damage and stress-response signaling in vitro while maintaining sufficient cell viability for transcriptional analysis. Finally, although statistically significant differences between BM- and UC-derived exosome treatments were limited, several gene expression patterns suggested that BM-derived exosomes may exert somewhat stronger modulatory effects on certain radiation-responsive pathways. These trends should be interpreted cautiously but may reflect differences in exosomal cargo composition between MSC sources that warrant further investigation in future studies.

To further elucidate the mechanisms by which BM- and UC-derived exosomes contribute to modulation of radiation-induced cellular responses, additional studies evaluating the functions of their differing proteomic and miRNA cargo may be particularly valuable. For example, it has been previously shown that UC-MSC exosomes are specifically enriched in certain miRNAs—miR-100, miR-146a, miR-21, miR-221, and miR-143—which were confirmed to promote vaginal epithelial cell proliferation and inhibit apoptosis through both miRNA overexpression and knockdown approaches [32]. This present study focused on the transcriptional effects of MSC-derived exosomes in a simple single-cell-type CRI model; future work integrating more functional assays and in vivo models will be important to determine whether these transcriptional changes translate into measurable improvements in DNA repair efficiency, oxidative stress burden, SASP factor accumulation, and long-term proliferative capacity. Such work will also help clarify the underlying mechanisms and evaluate the broader therapeutic and regenerative potential of MSC-derived exosomes in mitigating CRI and related aging-associated processes.

## Figures and Tables

**Table 1:** TaqMan Gene Expression Assays used for qPCR.

Gene	TaqMan Gene Expression Assay
*CDKN1A*	Hs00355782_m1
*CDKN2A*	Hs00923894_m1
*DDB2*	Hs03044949_m1
*FDXR*	Hs01031617_m1
*GADD45A*	Hs00169255_m1
*GAPDH*	Hs02786624_g1
*GDF15*	Hs00171132_m1
*H2AFX*	Hs00266783_s1
*IL-6*	Hs00174131_m1
*MKI67*	Hs04260396_g1
*RNF8*	Hs00187634_m1
*SESN1*	Hs00902782_m1
*SOD1*	Hs00533490_m1
*TNFAIP3*	Hs00234713_m1
*VEGFA*	Hs00900055_m1

## Data Availability

The original contributions presented in this study are included in the article. Further inquiries can be directed to the corresponding author.

## Abbreviations

The following abbreviations are used in this manuscript:

ADSC	Adipose-derived stem cell
BM	Bone marrow
CRI	Cutaneous radiation injury
FI	Functional interaction
HDF	Human dermal fibroblast
IR	Ionizing radiation
MSC	Mesenchymal stem cell
MSC-exo	Mesenchymal stem cell–derived exosome
NTA	Nanoparticle tracking analysis
SASP	Senescence-associated secretory phenotype
UC	Umbilical cord
